# Quantitative Diffusion-Weighted MR Imaging: Is There a Prognostic Role in Noninvasively Predicting the Histopathologic Type of Uveal Melanomas?

**DOI:** 10.3390/cancers15235627

**Published:** 2023-11-29

**Authors:** Pietro Valerio Foti, Corrado Inì, Giuseppe Broggi, Renato Farina, Stefano Palmucci, Corrado Spatola, Maria Chiara Lo Greco, Emanuele David, Rosario Caltabiano, Lidia Puzzo, Andrea Russo, Antonio Longo, Teresio Avitabile, Antonio Basile

**Affiliations:** 1 Department of Medical Surgical Sciences and Advanced Technologies “G.F. Ingrassia”—Radiology I Unit, University Hospital Policlinico “G. Rodolico-San Marco”, Via Santa Sofia 78, 95123 Catania, Italy; corrado.ini@gmail.com (C.I.); radfaro@hotmail.com (R.F.); spalmucci@sirm.org (S.P.); cor_spatola@hotmail.com (C.S.); mariachiaralg@gmail.com (M.C.L.G.); david.emanuele@yahoo.it (E.D.); basile.antonello73@gmail.com (A.B.); 2 NANOMED-Research Centre for Nanomedicine and Pharmaceutical Nanotechnology, University of Catania, 95125 Catania, Italy; 3 Centro di Ricerca Multidisciplinare “Chirurgia delle Sindromi Malformative Complesse della Transizione e dell’Età Adulta” (ChiSMaCoTA), Department of Medical Surgical Sciences and Advanced Technologies “G.F. Ingrassia”, University of Catania, 95123 Catania, Italy; 4 Section of Anatomic Pathology, Department of Medical Surgical Sciences and Advanced Technologies “G.F. Ingrassia”, University of Catania, Via Santa Sofia 78, 95123 Catania, Italy; giuseppe.broggi@gmail.com (G.B.); rosario.caltabiano@unict.it (R.C.); lipuzzo@unict.it (L.P.); 5 Department of Ophthalmology, University of Catania, Via Santa Sofia 78, 95123 Catania, Italy; andrearusso2000@hotmail.com (A.R.); antlongo@unict.it (A.L.); t.avitabile@unict.it (T.A.)

**Keywords:** eye (A01.456.505.420), uvea (A09.371.894), eye neoplasms (C04.588.364), uveal neoplasms (C04.588.364.978), melanoma (C04.557.465.625.650.510), magnetic resonance imaging (E01.370.350.825.500), diffusion magnetic resonance imaging (E01.370.350.825.500.150), biopsy, needle (E04.074.119), prognosis (E01.789), eye enucleation (E04.540.429)

## Abstract

**Simple Summary:**

Histologic type is an important prognostic determinant in the clinical workup of uveal melanoma (UM), with different histologic types being related to different metastatic risk and hence mortality rates. The diagnosis of UM is predominantly clinical, whereas biopsy, although capable of providing the histologic type, is not routinely performed due to its invasiveness and complications. Moreover, biopsy often provides contradictory results because of the heterogeneity of UMs. In this context, recently the interest has grown as regards to noninvasive biomarkers, which can represent alternative methods of prognostication. We verified whether magnetic resonance imaging and, in particular, one of its functional techniques, diffusion-weighted imaging, could be helpful in distinguishing the different histologic types of UMs and therefore play a prognostic role. Whilst negative, our preliminary results could represent a starting point for future research in the context of a patient-centered approach of healthcare.

**Abstract:**

Histopathologically, uveal melanomas (UMs) can be classified as spindle cell, mixed cell and epithelioid cell type, with the latter having a more severe prognosis. The aim of our study was to assess the correlation between the apparent diffusion coefficient (ADC) and the histologic type of UMs in order to verify the role of diffusion-weighted magnetic resonance imaging (DWI) as a noninvasive prognostic marker. A total of 26 patients with UMs who had undergone MRI and subsequent primary enucleation were retrospectively selected. The ADC of the tumor was compared with the histologic type. The data were compared using both one-way analysis of variance (ANOVA) (assessing the three histologic types separately) and the independent *t*-test (dichotomizing histologic subtypes as epithelioid versus non-epithelioid). Histologic type was present as follows: the epithelioid cell was *n* = 4, and the spindle cell was *n* = 11, the mixed cell type was *n* = 11. The mean ADC was 1.06 ± 0.24 × 10^−3^ mm^2^/s in the epithelioid cells, 0.98 ± 0.19 × 10^−3^ mm^2^/s in the spindle cells and 0.96 ± 0.26 × 10^−3^ mm^2^/s in the mixed cell type. No significant difference in the mean ADC value of the histopathologic subtypes was found, either when assessing the three histologic types separately (*p* = 0.76) or after dichotomizing the histologic subtypes as epithelioid and non-epithelioid (*p* = 0.82). DWI-ADC is not accurate enough to distinguish histologic types of UMs.

## 1. Introduction

Uveal melanoma (UM) is a malignant neoplasm arising from neural crest-derived melanocytes of the uvea, which is the vascular pigmented layer of the eye [1,2]. The diagnosis of UM is primarily clinical, which is based on noninvasive ophthalmological techniques (indirect ophthalmoscopy) and is associated, where necessary, with supplementary ophthalmological imaging modalities (fundus fluorescein angiography, optical coherence tomography and A-mode scan and B-mode scan ultrasonography) and radiological imaging tools (magnetic resonance imaging MRI) [3,4]. A biopsy is not routinely performed for diagnostic purposes [5,6]; however, it can be used in the case of lesions with atypical appearance and, above all, for prognostication [7,8,9,10,11,12]. In the last few decades, a wide variety of prognostic parameters have been detected and investigated: tumor location and dimensions, histologic type, mitotic count, pigmentation, infiltrating lymphocytes and genetic alterations [13,14,15,16]. As for histopathologic features, UM encompasses three histopathological subtypes: epithelioid cell type, spindle cell type and mixed type, accounting for approximately 3–5%, 40% and 50% of all UMs, respectively [17,18]. Patients with the epithelioid cell type have a worse prognosis than patients with the spindle cell type, with the former being associated with a higher likelihood of metastasis development and a higher mortality rate; in particular, the 15-year mortality rate is 75% for epithelioid, 20% for spindle cell type and 60% for mixed type [4,17,18,19,20]. Although the etiopathogenesis of UMs is still not entirely clear, many studies have shed light on the role of cytogenetic and transcriptional abnormalities both in the carcinogenesis process and in metastasization. Chromosome 1p loss, 3 loss, 6q loss and 8q gain are indicative of a poor prognosis. Transcriptomic features can identify two different classes of genetic signature: Class I and Class II tumors, with low metastatic risk and high metastatic risk, respectively [1,17,21,22,23,24,25,26,27].

Prognostic fine-needle aspiration biopsy (FNAB) may be useful to identify patients at high risk for liver metastasis. These patients may benefit from a dedicated schedule of systemic closer surveillance for metastases, early treatment of focal liver lesions and enrollment into adjuvant systemic therapy clinical trials [28,29]. FNAB requires local or general anesthesia and, although over the years this has become an effective and safe procedure in experienced hands [19,30], it is not without complications [10,19]. FNAB-related complications can be distinguished as minor and major. Minor complications (resolving on their own) are relatively common: minor vitreous or subretinal hemorrhage, retinal detachment and transient localized perilesional bleeding. On the other hand, major complications (needing intervention) are rare (1–5.9%): persistent/recurrent vitreous or subretinal hemorrhage, submacular hemorrhage, retinal perforation and rhegmatogenous retinal detachment [9,10,19,28,29,31]. Neoplastic cell seeding along the needle tract is quite common; however, intraocular biopsy does not seem to be related to an increased metastatic risk to date [10,28,32], and cancer recurrence at the needle insertion site has not been documented [29]. When performing FNAB, it should be remembered that sampling errors are still not infrequent because of the histopathologic and cytogenetic heterogeneity of UMs [5,10,33,34,35,36,37,38,39,40].

In this scenario, a noninvasive biomarker, capable of providing useful information to stratify patients undergoing eye-saving treatments, is highly desirable. MRI has been applied in the pretreatment assessment of UMs, allowing for more accurate treatment planning [41]. In the last few years, diffusion-weighted magnetic resonance imaging (DWI) has been widely used in the field of oncology [42] and, in particular, in the study of UMs [24,43]. In vivo, the diffusion of water molecules is affected by various cellular microstructures such as macromolecules, intracellular organelles and especially cell membranes [44]. A natural question arises; namely, whether DWI, and, in particular, the apparent diffusion coefficient (ADC) value, which is the quantitative parameter of DWI, could make a contribution in an attempt to identify differences with respect to cell type in UMs.

Therefore, the aim of our study was to retrospectively assess the correlation between ADC values and histopathologic type of UMs in order to verify the clinical value of DWI as a noninvasive prognostic imaging biomarker.

## 2. Materials and Methods

### 2.1. Patient Population and Selection Criteria

The study design was a retrospective, consecutive, observational, single-center case series. The Anatomic Pathology Section database and the Radiology Unit database of orbital/ocular lesions, generated and continuously updated by consecutive review of MRI examinations from 2016 to 2023, were queried. Forty-eight patients, who had undergone an MR examination and subsequent surgical enucleation for UM between September 2016 and June 2023, were retrospectively identified. Our study was approved by the institutional ethics committee of the University of Catania for studies involving humans (Comitato Etico Catania 1, Protocol N. 24114, approved on 21 May 2021). The inclusion criteria were as follows: clinical diagnosis of UM; MR examination of the brain and orbits performed in the radiology department of our hospital prior to ocular enucleation; primary enucleation within two weeks of the MRI examination; final histologic diagnosis of UM. The exclusion criteria were as follows: incomplete MR protocol; poor diagnostic quality of the MR examination; patients undergoing loco-regional therapy prior to enucleation; enucleation performed at hospitals other than our own; more than two weeks between the MR examination and enucleation; lesions with a prominence ≤3 mm. The study was conducted in accordance with the World Medical Association’s Code of Ethics (Declaration of Helsinki) concerning ethical principles for biomedical research involving human beings. All the patients gave written informed consent for the publication of their data for scientific purposes.

### 2.2. MRI Protocol

The MR imaging protocol used to study patients with UM made use of a closed-configuration superconducting 1.5-T MRI scanner (Signa HDxT, GE Healthcare, Milwaukee, WI, USA) with 57.2 mT/m gradient strength and 120 T/m/s slew rate, and an 8-channel high-resolution neurovascular phased-array coil equipped with array spatial sensitivity technique (ASSET) parallel acquisition. All patients underwent MR examination of the brain and orbits. The MR imaging protocol of the orbit included anatomical and functional sequences and is summarized in Table 1.

### 2.3. Histopathology

All surgical samples were 4% formalin-fixed and paraffin-embedded; eight sections comprising the tumor, the optic nerve and the pupil were cut to 4–5 micron and routinely stained with hematoxylin and eosin. Two pathologists (R.C. and G.B.) with expertise in UM histopathology reviewed all tumor specimens with no information on patients’ clinical and radiologic data. The modified Callender classification was adopted to classify all cases [45].

### 2.4. Image Analysis

In accordance with the study design, MR images were assessed by two radiologists (P.V.F. and C.I., where the former had 11 years of professional experience and the latter had 5 years of professional experience in the field of orbital imaging).

The quality of DW images was firstly estimated with a three-point scale by the two radiologists in consensus, as previously described: (1) minor distortion and/or motion artifacts, mass clearly visible; (2) moderate distortion and/or motion artifacts, mass blurred; (3) major distortion and/or motion artifacts, mass not visible [46].

The quantitative estimation of the apparent diffusion coefficient (ADC) of the tumors on DW images was performed in consensus by way of a dedicated workstation with diffusion analysis software (Advantage Windows version 4.6, General Electric Medical Systems, Milwaukee, WI, USA).

Briefly, on the DW sequence, the radiologists selected the image in which the tumor showed the greatest extent and placed an oval region of interest (ROI), including almost the maximum surface area of the lesion, while at the same time excluding distortion artifacts and the edge of the lesion with the aim of avoiding partial volume effects; the ROI was mechanically copied on the corresponding ADC map. T1- and T2-weighted images were synchronized with DW images and were thoroughly taken into account to identify tumor morphology and boundaries and to exclude macroscopically detectable necrotic or cystic components [47]. The measurement was repeated three times, resulting in a total of three subreadings for each lesion; the values of the three subreadings were averaged and the mean ADC value was obtained for each tumor and then used in the final analysis. Possible lesions with a prominence ≤3 mm, as mentioned above, were excluded from the quantitative DWI assessment due to an untrustworthy ADC evaluation [48].

The tumor prominence (TP) (estimated including the scleral thickness) and the tumor largest basal diameter (LBD) were assessed both at MRI examinations and at histopathology.

### 2.5. Statistical Analysis

Statistical analysis was performed by means of *Social Science Statistics* [49]; the significance level was established at *p* ≤ 0.05. ADC values are presented as the mean ± standard deviation. The ADC value of the tumor was compared with the histologic type. Statistical differences of the ADC values among the three histologic types were assessed using one-way analysis of variance (ANOVA). Furthermore, the histologic type was dichotomized as epithelioid versus non-epithelioid cell type (the latter including spindle cell type and mixed cell type), and the data were compared by using the independent *t*-test (unpaired *t*-test, two-tailed hypothesis).

Statistical differences of the tumor dimensions (both TP and LBD) obtained at MRI examinations and at histopathology were assessed using the independent *t*-test. Statistical differences of the TP and of LBD (measured at MRI) among the three histologic types were assessed using one-way analysis of variance (ANOVA).

Correlation between the ADC value and TP on MRI was tested by means of Pearson’s r.

## 3. Results

### 3.1. Study Population

Based on the abovementioned inclusion and exclusion criteria, 28 patients with UM having undergone MR examination and subsequent primary enucleation within two weeks were retrospectively selected for eventual inclusion in the study. Of these, two were excluded because surgical treatment was performed in a hospital other than our own. Therefore, the final patient cohort for the histologic and MRI assessment consisted of 26 patients (15 males, 11 females, mean age of 61 years, range of 35–86 years). The demographic data of the population included in the study are summarized in Table 2.

**Figure 1:**
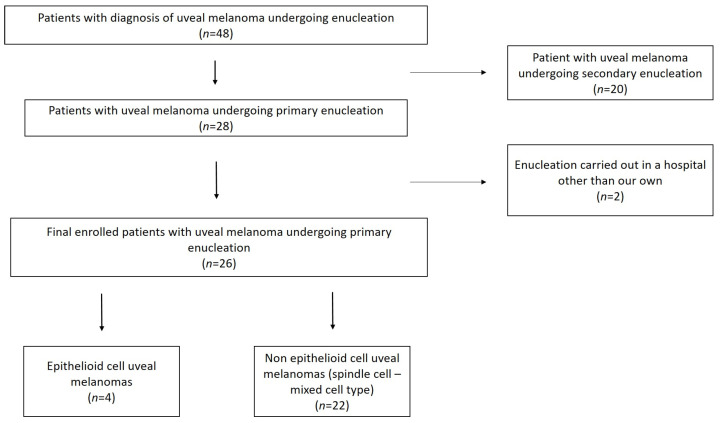
Flow diagram displays the patient selection process.

Patients included in our research belong to an overall group of 48 patients with UM who underwent enucleation (both primary and secondary) throughout the time interval of the study (Figure 1).

### 3.2. Histopathologic Findings

In all patients, UMs originated from the choroid. In 4/26 (15.4%) patients, UM invaded the ciliary body and, in 3/26 (11.5%) patients, the optic nerve was involved by the tumor. One patient presented extrascleral invasion.

Histopathologically, 4/26 (15.4%) patients were affected by the epithelioid cell type, 11/26 (42.3%) patients by the spindle cell type and 11/26 (42.3%) patients by the mixed cell type UM.

A total of 20/26 (77%) patients had a retinal detachment at the time of diagnosis, and, of these, 10 were hemorrhagic.

### 3.3. ADC Measurement and Relationship with Histologic Type

**Figure 2:**
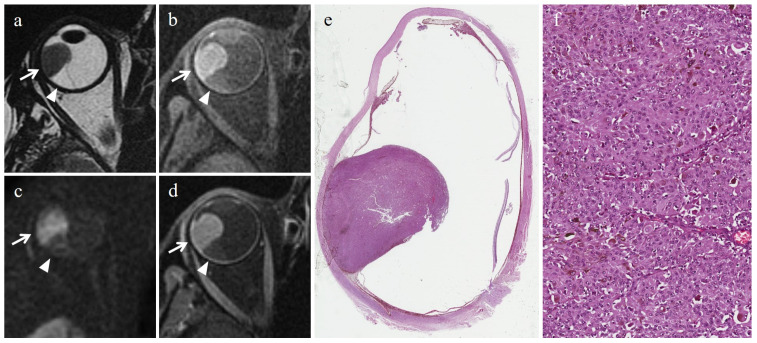
Epithelioid cell UM. A 65-year-old female patient with a choroidal melanoma of the right eye. Axial (**a**) T2-weighted turbo spin-echo and (**b**) fat-suppressed T1-weighted images show a dome-shaped intraocular lesion along the lateral aspect of the globe (white arrows). Along the posterior aspect of the lesion, a serous retinal detachment is detectable on both T2- and T1-weighted images (white arrowheads). On (**c**) axial DW image (b = 1000 s/mm^2^) the mass exhibits restricted diffusion with high signal intensity (white arrow), conversely from the retinal detachment that does not show restricted diffusion (white arrowhead). On (**d**) the axial contrast-enhanced fat-suppressed T1-weighted image, the tumor is enhanced (white arrow); on the other hand, the retinal detachment is not enhanced (white arrowhead). (**e**) Histological examination: low magnification showing a dome-shaped mass protruding into the posterior segment of the eye and inducing a retinal detachment (H&E, original magnification 25×). (**f**) High magnification showing the epithelioid cell uveal melanoma composed of nests of polygonal cells with large eosinophilic cytoplasm and rounded nuclei with coarse chromatin (H&E; original magnification 300×).

**Figure 3:**
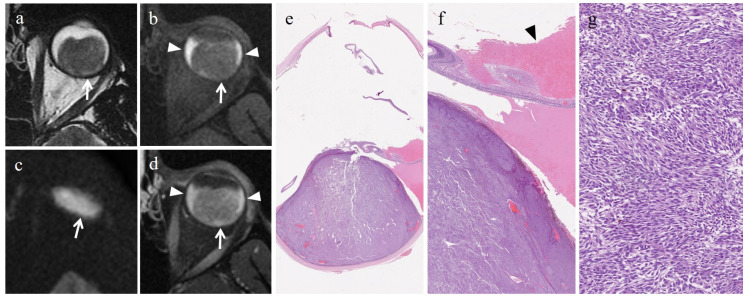
Spindle cell UM. A 39-year-old male patient with a choroidal melanoma of the left eye. Axial (**a**) T2-weighted turbo spin-echo and (**b**) fat-suppressed T1-weighted images display a bulky intraocular mass along the posterior aspect of the globe (white arrows). Axial (**c**) DW image (b = 1000 s/mm^2^) and (**d**) contrast-enhanced fat-suppressed T1-weighted image. The lesion demonstrates restricted diffusion (white arrow in (**c**)) and moderate enhancement (white arrow in (**d**)). Note the hemorrhagic retinal detachment on both sides of the mass are more obvious in the T1-weighted images (white arrowheads in (**b**,**d**)). (**e**) Histological examination: low magnification showing a poorly-pigmented tumor protruding into the posterior segment of the eye (H&E, original magnification 25×). (**f**) Histological detail (higher magnification) revealing a tumor-induced hemorrhagic retinal detachment (black arrowhead) (H&E, original magnification 100×). (**g**) Higher magnification showing the tumor with spindle cell morphology consisting of spindle-shaped cells with fusiform nuclei arranged in short intersecting fascicles (H&E; original magnification 300×).

In all patients, DW images had sufficient quality for quantitative assessment; in particular, the quality of DW images was judged as 1 in 23 patients, 2 in 3 patients and 3 in 0 patients. The overall ADCs of UMs ranged from 0.69 × 10^−3^ mm^2^/s to 1.59 × 10^−3^ mm^2^/s, with a mean of 0.98 × 10^−3^ mm^2^/s and a standard deviation of 0.22 × 10^−3^ mm^2^/s. The epithelioid cell type presented ADC values ranging from 0.73 × 10^−3^ mm^2^/s to 1.26 × 10^−3^ mm^2^/s, with a mean of 1.06 × 10^−3^ mm^2^/s and a standard deviation of 0.24 × 10^−3^ mm^2^/s (Figure 2). The spindle cell type showed ADC values ranging from 0.69 × 10^−3^ mm^2^/s to 1.29 × 10^−3^ mm^2^/s, with a mean of 0.98 × 10^−3^ mm^2^/s and a standard deviation of 0.19 × 10^−3^ mm^2^/s (Figure 3). The mixed cell type showed ADC values ranging from 0.73 × 10^−3^ mm^2^/s to 1.59 × 10^−3^ mm^2^/s, with a mean of 0.96 × 10^−3^ mm^2^/s and a standard deviation of 0.26 × 10^−3^ mm^2^/s. Non-epithelioid cell type (including spindle cell and mixed cell type jointly) presented ADC values between 0.69 × 10^−3^ mm^2^/s and 1.59 × 10^−3^ mm^2^/s, with a mean of 0.97 × 10^−3^ mm^2^/s and a standard deviation of 0.22 × 10^−3^ mm^2^/s. The mean ADC values of the enrolled population of the study are summarized in Table 3.

One-way ANOVA of the ADC values revealed no significant differences among the three histopathologic types (F2, 23 = 0.27; *p* = 0.76). Moreover, the unpaired *t*-test showed that there was no statistically significant difference between the mean ADC value of epithelioid cell type and non-epithelioid cell type (mean ADC 1.06 × 10^−3^ mm^2^/s and 0.97 × 10^−3^ mm^2^/s; SD 0.24 and 0.22 × 10^−3^ mm^2^/s; t(24) = 0.22, *p* = 0.82) (Table 4).

### 3.4. Tumor Dimensions and Relationship with Histopathologic Type

Overall, the TP measured at MRI ranged from 4 mm to 16 mm, with a mean of 10.2 mm and a standard deviation of 3.1 mm. The TP measured at histopathology ranged from 3 mm to 15 mm, with a mean of 9.2 mm and a standard deviation of 3 mm (Table 5).

The LBD measured at MRI ranged from 10 mm to 23 mm, with a mean of 15.4 mm and a standard deviation of 3.4 mm. The LBD measured at histopathology ranged from 7 mm to 20 mm, with a mean of 14.1 mm and a standard deviation of 3.4 mm (Table 5).

The unpaired *t*-test showed that there was no statistically significant difference between the TP measured at MRI examinations and at histopathology (t(24) = 0.45, *p* = 0.64). The unpaired *t*-test showed that there was no statistically significant difference between the LBD measured at MRI examinations and at histopathology (t(24) = 0.53, *p* = 0.6).

One-way ANOVA of the TP measured at MRI revealed no significant differences among the three histopathologic types (F2, 23 = 0.03; *p* = 0.96). One-way ANOVA of the LBD measured at MRI revealed no significant differences among the three histopathologic types (F2, 23 = 0.2; *p* = 0.81).

**Figure 4:**
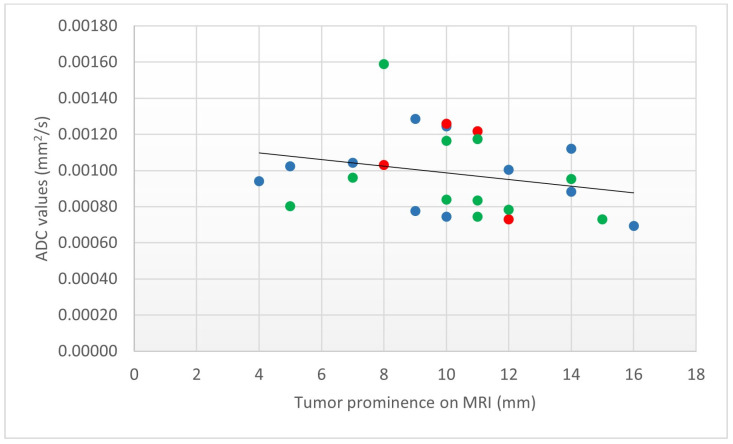
Correlation between the ADC value and TP on MRI. Scatterplot (ADC vs. TP) demonstrating that smaller TP tends to have higher ADC values and vice versa (r(24) = −0.87, *p* < 0.0001). The markers are coded for cell type as follows: ● epithelioid cell type, ● mixed cell type and ● spindle cell type.

Lastly, a relationship between the tumor ADC value and the TP on MRI was analyzed using the Pearson correlation coefficient: a significant negative correlation was found (r(24) = −0.87, *p* < 0.0001) (Figure 4).

## 4. Discussion

In our study, we assessed the usefulness of DW-MRI in discriminating between the different histologic types of UMs. UMs are a heterogeneous group of neoplasms encompassing three histopathological subtypes and different genetic profiles [1]. Histopathological subtyping provides productive information for patient management and, although it is generally agreed that molecular genetic testing overrules clinical features for the prognostic stratification, the detection of the histopathological subtype of UM is still key for predicting metastasis-related mortality [1,17,50,51].

In patients undergoing eye-saving treatments, histologic type can be identified through FNAB; nevertheless, this procedure is not routinely performed in the initial diagnostic assessment of patients with UM. The success rate of FNAB is connected to the capability of achieving sufficient cellularity for histopathological and genetic analysis and this varies, according to different authors, ranging from 50% [52] to 98% [1,19,53,54] and it is based on several factors: tumor location, FNAB approach, surgical technique, tumor dimensions, surgeon experience and molecular tests used [19,28,29,54,55,56]. In this regard, the tumor structure should also be taken into consideration. Bagger et al. reported on the difficulty of obtaining sufficient material in a lesion with a hard texture [10]. In the same way, tumors containing wide necrotic areas may also be difficult to biopsy and may need various needle passes to obtain sufficient material [28]. Tumor heterogeneity is another key factor we have to take into account when it comes to UMs. In this regard, it should be noted that the agreement between the histopathological classification of the tumor based on the biopsy and on the histologic analysis of the entire tumor after enucleation is relatively moderate (remaining at around 60%), because of the cytological heterogeneity of UMs [10,33]. Jensen et al., first, and Bagger et al., successively, reported an agreement of 61% and 59.1%, respectively [10,33]. Moreover, chromosomal aberrations may have an inhomogeneous distribution within the lesion, thus making biopsy not representative of the whole tumor even from a genetical point of view [5,34,35,36,37,38,39]. Jaarsma-Coes et al., in addition, recently confirmed the inhomogeneous distribution of MRI signal intensity features (T1 values), and, most importantly, of perfusion metrics within UMs [40].

In this context, biopsy findings should be interpreted with caution; moreover, a close cooperation between the ophthalmic surgeon and the cytopathologist, with perioperative tissue confirmation by the latter, is pivotal in order to obtain a successful biopsy that is adequate for both histopathologic examination and molecular analysis [1,10,57]. Therefore, as a result of the invasiveness, of the potential risks and of the aforesaid shortcomings of the bioptic procedure, interest has grown regarding noninvasive biomarkers, which can mirror the neoplastic structure as a whole.

In the last two decades, DWI has been widely applied to investigate several pathological conditions in numerous anatomical regions, so its clinical applications have been constantly increasing [58,59,60,61,62,63]. The usefulness of DWI in the study of eye tumors, and in particular of UM, has been debated by various authors [64,65]. Sepahdari et al. postulated the potential clinical role of DWI in characterizing indeterminate orbital masses [66,67,68]. Erb-Eigner et al. and Ferreira et al. demonstrated the capability of DWI in differentiating ocular tumors, in particular choroidal melanoma, from retinal detachment [43,69]. The role of the ADC value in detecting and predicting the treatment response of patients with UMs undergoing proton beam radiotherapy has been demonstrated [46,70]. Despite everything, the correlation between prognostic markers and functional quantitative MRI parameters is a relatively unexplored area of research. Only recently, Ferreira et al. first correlated prognostic markers of UMs with functional MRI features, finding a significant correlation between the ADC value and tumor prominence [5].

However, the biophysical mechanisms underpinning water diffusivity changes and, in particular, the relationship between the tissue structure and the DWI signal is still not fully understood [71].

In biological tissues, water diffusion is inhomogeneous and non-Gaussian, since it can be hindered by tissue compartments and by the presence, the intactness, the permeability and even the orientation of cell membranes. Specifically, tissues characterized by heightened nuclear/cytoplasmic ratio and high cellularity tend to demonstrate restricted diffusion [44,71,72,73,74].

**Figure 5:**
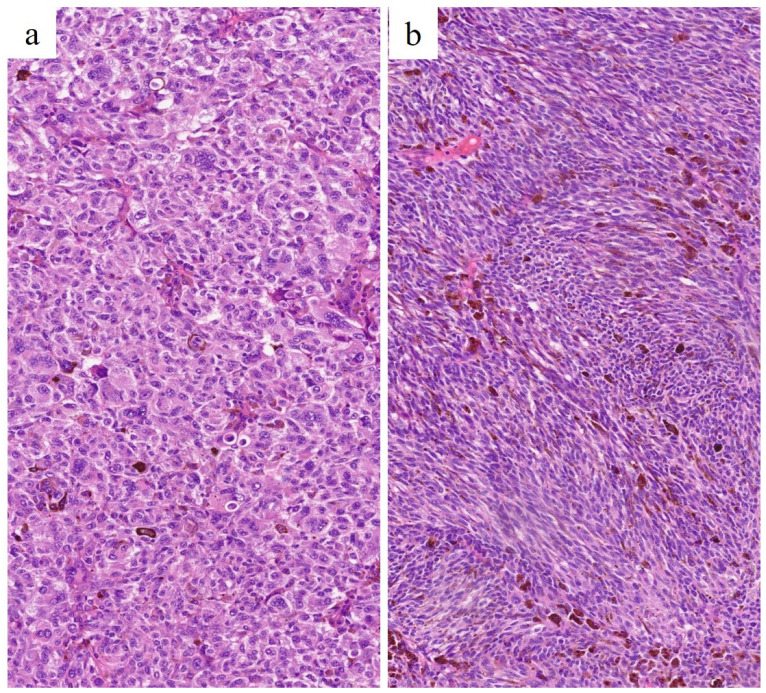
Histological features of UMs. (**a**) Epithelioid cell UM. Large polygonal cells with large eosinophilic cytoplasm and rounded nuclei with coarse chromatin are seen (H&E, original magnification 300×). (**b**) Spindle cell UM. Fusiform cells with scant elongated cytoplasm, ovoid nuclei and high nuclear-to-cytoplasmic ratio, arranged in short intersecting fascicles, are seen (H&E, original magnification 300×).

Histological types of UM differ in a number of ways. Epithelioid cell UM exhibits large and polygonal cells with a low nucleus/cytoplasm ratio that is characterized by rounded nuclei with coarse chromatin and plentiful eosinophilic cytoplasms. They show a trend to discohesion. On the other hand, the spindle cell type is composed of cells with elongated morphology, ovoid nuclei, scant eosinophilic cytoplasms and high nuclear-to-cytoplasmic ratio. They are thickly arranged in short intersecting fascicles, demonstrating a cohesive pattern. Mitotic count is usually higher in epithelioid tumors, whereas it is inconspicuous in spindle cell types [1,17,75] (Figure 5). On the basis of the above, we wondered whether the underlying tissue geometry of the different histopathological types of UM could affect water diffusivity and induce quantitatively measurable changes in ADC values, and, therefore, whether the ADC could represent a surrogate indicator for the microstructural complexity of the tissue microarchitecture [76].

In our case series, different histological types were distributed as follows: epithelioid cell type as 4/26 (15.4%), spindle cell type as 11/26 (42.3%) and mixed type as 11/26 (42.3%). Our data are roughly consistent with those from the literature, according to which the epithelioid histotype is the least common [17,18]. The overall mean ADC (SD) of UMs was 0.98 ± 0.22 × 10^−3^ mm^2^/s. This data are substantially in agreement with those obtained by other authors [5,67,69] and by our group in earlier studies [46,70].

In our case series, we found no significant difference in the mean ADC value of the histopathologic subtypes, either when assessing the histologic types separately into three distinct groups (*p* = 0.76) or after dichotomizing the histologic subtypes as epithelioid and non-epithelioid cell type (*p* = 0.82). The explanation of such a result might lie in the histological complexity of the different morphological subtypes of UMs. As we know, the ADC value is affected by various factors. Some features of epithelioid cells (i.e., abundant cytoplasm, low nuclear-to-cytoplasmic ratio, discohesive pattern) would tend to increase the ADC value; nevertheless, other variables (i.e., many mitotic figures) contribute to reduce the ADC value. The same happens for spindle cells, where drivers that tend to lower the ADC (i.e., scant cytoplasm, high nuclear-to-cytoplasmic ratio, cohesive pattern) and other determinants (i.e., few mitoses) that raise the ADC value coexist (Figure 5). Probably because of the aforementioned reasons, in our case series, epithelioid tumors showed an ADC value only slightly, but not significantly, higher than that of spindle cells tumors. To this, it must be added that the order of magnitude of DWI measurements is far greater than the cell size, and the ADC value is just an indicator of how much water molecules can spread; furthermore, the ADC value is only one of the DWI-derived metrics [76]. The ADC measurement derived from traditional DW sequences is burdened with some drawbacks, since it is obtained through a monoexponential model and is influenced by microcirculation perfusion. On the other hand, another DWI-based quantitative parameter, such as intravoxel incoherent motion (IVIM)-DWI, uses a biexponential model, thus discriminating information concerning pure water molecule diffusion from those regarding microcirculation perfusion and therefore separating the assessment of diffusion and perfusion in biological tissues [77,78,79,80,81].

In our case series, the tumor dimensions (TP and LBD) obtained at MRI examinations were overall larger than those obtained at histopathology. As previously reported by Ferreira et al., this was due to tumor shrinkage occurring as a result of the fixation process of the histopathological samples [5]. Nevertheless, in confirmation of the feasibility of MRI measurements, no statistically significant difference was found between the tumor dimensions assessed with MRI and with histopathology.

No significant differences among the three histopathologic types were found in terms of TP and LBD.

We found a significant negative correlation between the tumor ADC value and the TP on MRI. This means that tumors with smaller TP tends to have higher ADC values and, vice versa, larger tumors tend to demonstrate lower ADC values (Figure 4). Our result is in agreement with the one recently reported by Ferreira et al. [5]. The reason for such a result could be twofold. In small lesions, the partial volume effect with the vitreous tends to be more evident than in large tumors, thus contributing to increase ADC values. Furthermore, it is reasonable that tumors with high cellularity (and therefore with lower ADC) may grow faster and hence demonstrate greater dimensions at diagnosis.

Despite the predominantly negative results of our study, the possible advantages of MRI in the study of such a heterogeneous neoplasm as UM remain undeniable. The biopsy, indeed, in addition to being an invasive procedure, provides an overly partial depiction of the tumor; on the other hand, MRI has the potential to allow for a more global assessment of the morpho-functional abnormalities of the lesion [5].

As for the MRI protocol, DW sequences deserve a mention. Conventional DWI uses single-shot echo-planar imaging (SS-EPI) that decreases the acquisition time, thus reducing motion artifacts. Nevertheless, because of the EPI trajectory, this pulse sequence is particularly affected by B0-inhomogeneities and this implies a number of artifacts, such as geometric distortion and image blurring, especially on the border between tissues with different susceptibilities (i.e., air/bone and air/tissue interfaces). SS-EPI sequences particularly suffer from large field of view (FOV); on the other hand, image quality can be improved by reducing the FOV in the phase-encoding direction of the EPI read-out, thus cutting down off-resonance-induced artifacts [82]. On the basis of the relatively small FOV used in the study of the orbit and of the reduced acquisition time, in our protocol we used EPI DW sequences. Our choice is certainly questionable. Various authors prefer, indeed, non-EPI DWI sequences; these need longer acquisition times than EPI DWI, but they are less prone to susceptibility artifacts and geometric distortion, which is a very relevant factor when studying orbital structures [5,43].

There are various limitations in our study. The retrospective design is an intrinsic limitation. The main limitation regarding our case series is its heterogeneity that is related to the noticeable amount of difference of the components of the three groups of patients (epithelioid (*n* = 4), spindle cell (*n* = 11), mixed type (*n* = 11)), even if the relative rarity of epithelioid UMs is widely reported in the literature. On the other hand, the overall number of patients (*n* = 26) is only seemingly small, since UM is a rare pathology and enucleation (representing our standard of reference) has become decreasingly frequent owing to the constant development of globe-salvaging approaches [83,84]. In our study, we took into account the ADC value, whereas we investigated the role neither of other potential quantitative parameters obtained from DWI (intravoxel incoherent motion diffusion-weighted imaging IVIM-DWI parameters, diffusion kurtosis parameter) that give different information from that provided by the ADC nor of other functional MRI techniques such as perfusion-weighted imaging [48]. Other limitations are related to our MR equipment (field strength 1.5T, 8-channel neurovascular coil), which are less powerful than other state-of-the-art MR scanners (field strength ≥3T and 32 channels or dedicated eye/orbit coil). The EPI-based DW sequence of our protocol was hampered by B0 inhomogeneity artifacts, as mentioned above. Furthermore, our DW sequence had a slice thickness of 4 mm and was acquired in the axial plane rather than in a tumor-specific orientation. Theoretically, this would have led to partial volume effects (as suggested by the negative correlation between the tumor ADC value and TP); nevertheless, we must not forget that our cohort consisted exclusively of UMs having undergone primary enucleation and, for this reason, exhibited predominantly large tumors. In particular, 23/26 (88%) lesions had a prominence >5 mm and 17/26 (65%) tumors showed a prominence ≥10 mm. Lastly, we confined ourselves to assessing the relationship between the histopathologic type and the ADC value, and we did not take into account the role of other prognosticators that were equally, if not more important, such as molecular abnormalities including chromosome 3-status, chromosome 8-status and BAP1-status, because of the current unavailability of adequate molecular investigation tools in our laboratory.

## 5. Conclusions

The ongoing changeover from evidence-based medicine to a more patient-centered approach along with the drawbacks of biopsy, partially related to the heterogeneity of UMs, account for the search for noninvasive methods of prognostication capable of predicting the clinical behavior of the tumor.

In our study, we found no significant difference in the mean ADC value of epithelioid, spindle cell type and mixed type of UMs, and therefore we must deduce that the ADC value does not sufficiently reflect the cytoarchitectonic features of the three different histologic subtypes.

However, although the ADC value demonstrated itself as an unreliable biomarker to detect cell-type-related differences in UMs, the research into noninvasive surrogate indicators for histopathologic structures and even for molecular biomarkers is only at the beginning. Future studies should focus on functional/quantitative MRI-derived biomarkers to be used as surrogate indicators for microstructural complexity and molecular expression in order to provide a contribution to stratify patients into different prognostic classes, thereby tailoring follow-up measures and adjuvant therapies. In particular, the possibility of also correlating the ADC value with other molecular prognostic factors of UMs could offer one of the most intriguing perspectives in future studies.

## Figures and Tables

**Table 1:** MR imaging protocol of the orbit. The table illustrates the technical parameters of MR sequences. Fat-suppressed T1-weighted sequences were acquired both pre- and post-intravenous administration of gadolinium-based contrast medium (gadoterate meglumine gd-DOTA, Dotarem, Guerbet, Roissy, France) dosage of 0.1 mmol/kg body weight.

MRI Protocol	T2W FSE	T2W FSE STIR	T1W FSE	T1W FSE Fat Sat	DWI SE EPI
Acquisition plane	axial, coronal	axial, coronal	axial, coronal	axial, coronal	axial
Repetition time/Echo time (ms)	3220/120	3700/50	550/14.9	450/15.1	4800/89.9
Flip angle	90°	90°	90°	90°	90°
Echo train length	19	12	2	2	-
N. of averages	4	3	3	2	8
Slice thickness (mm)	3	3	3	3	4
Interslice gap (mm)	0.3	0.3	0.3	0.3	0.4
Field of view (mm)	160 × 160	160 × 160	160 × 160	160 × 160	200 × 200
Matrix	352 × 256	256 × 256	256 × 224	256 × 256	192 × 192
Frequency direction	Superior to inferior	Anterior to posterior	Right to left	Right to left	Right to left
b-value (s/mm^2^)	-	-	-	-	0–1000
Scan time	3 min 20 s	4 min 12 s	3 min 30 s	3 min 30 s	3 min 40 s

T1W = T1-weighted, T2W = T2-weighted, FSE = fast spin-echo, STIR = short tau inversion recovery, fat sat = fat saturation (frequency-selective fat saturation), DWI = diffusion-weighted imaging, SE = spin-echo and EPI = echoplanar imaging.

**Table 2:** Demographic data of the enrolled population.

Patient	Gender	Age	Eye	Tumor Location	Histologic Type
1	Male	55	Left	Choroid	Epithelioid cell type
2	Female	55	Right	Choroid	Spindle cell type
3	Female	80	Right	Choroid	Epithelioid cell type
4	Female	81	Left	Choroid	Spindle cell type
5	Male	77	Right	Choroid and ciliary body	Spindle cell type
6	Male	70	Right	Choroid and ciliary body	Mixed cell type
7	Male	40	Left	Choroid	Spindle cell type
8	Male	54	Right	Choroid and ciliary body	Mixed cell type
9	Female	69	Left	Choroid	Spindle cell type
10	Female	79	Left	Choroid	Mixed cell type
11	Male	64	Left	Choroid	Spindle cell type
12	Male	49	Left	Choroid	Mixed cell type
13	Female	72	Left	Choroid	Mixed cell type
14	Female	47	Left	Choroid	Mixed cell type
15	Male	70	Left	Choroid	Spindle cell type
16	Female	36	Right	Choroid and ciliary body	Spindle cell type
17	Male	35	Left	Choroid	Epithelioid cell type
18	Female	69	Right	Choroid	Mixed cell type
19	Female	65	Right	Choroid	Epithelioid cell type
20	Male	39	Left	Choroid	Spindle cell type
21	Male	61	Right	Choroid	Mixed cell type
22	Male	40	Right	Choroid	Spindle cell type
23	Male	61	Right	Choroid	Mixed cell type
24	Female	86	Left	Choroid	Mixed cell type
25	Male	72	Left	Choroid	Spindle cell type
26	Male	66	Left	Choroid	Mixed cell type

**Table 3:** Histologic types and quantitative measurements.

Patient	Histologic Type	Mean ADC Values (×10^−3^ mm^2^/s)	Tumor Prominence (mm) * at MRI	Tumor Largest Basal Diameter (mm) at MRI
1	Epithelioid cell type	0.00103	8	20
2	Spindle cell type	0.00102	5	10
3	Epithelioid cell type	0.00122	11	12
4	Spindle cell type	0.00100	12	16
5	Spindle cell type	0.00069	16	21
6	Mixed cell type	0.00074	11	23
7	Spindle cell type	0.00124	10	14
8	Mixed cell type	0.00116	10	14
9	Spindle cell type	0.00094	4	14
10	Mixed cell type	0.00083	11	12
11	Spindle cell type	0.00104	7	18
12	Mixed cell type	0.00117	11	16
13	Mixed cell type	0.00095	14	14
14	Mixed cell type	0.00159	8	15
15	Spindle cell type	0.00112	14	15
16	Spindle cell type	0.00129	9	12
17	Epithelioid cell type	0.00126	10	19
18	Mixed cell type	0.00096	7	16.5
19	Epithelioid cell type	0.00073	12	10
20	Spindle cell type	0.00088	14	19
21	Mixed cell type	0.00078	12	19
22	Spindle cell type	0.00074	10	12.5
23	Mixed cell type	0.00080	5	12
24	Mixed cell type	0.00073	15	18
25	Spindle cell type	0.00078	9	12.5
26	Mixed cell type	0.00084	10	15

* Measured including the scleral thickness.

**Table 4:** ADC measurement and relationship with histologic type.

Enrolled Population	Mean ADC Values ± (SD) (×10^−3^ mm^2^/s)
Patients with UM (tot.) (*n* = 26)	0.98 ± 0.22
Epithelioid cell type UM (*n* = 4)	1.06 ± 0.24
Spindle cell type UM (*n* = 11)	0.98 ± 0.19
Mixed cell type UM (*n* = 11)	0.96 ± 0.26
Non-epithelioid cell type UM (*n* = 22)	0.97 ± 0.22
One-way ANOVA	F2, 23 = 0.27; *p* = 0.76
Unpaired *t*-test	*t*(24) = 0.22, *p* = 0.82

**Table 5:** Tumor dimensions measured at MRI and at histopathology.

Patient	Histologic Type	Tumor Prominence (mm) * at MRI	Tumor Prominence (mm) at Histology	Tumor Largest Basal Diameter (mm) at MRI	Tumor Largest Basal Diameter (mm) at Histology
1	Epithelioid cell type	8	13	20	20
2	Spindle cell type	5	5	10	7
3	Epithelioid cell type	11	8	12	13
4	Spindle cell type	12	10	16	14
5	Spindle cell type	16	15	21	20
6	Mixed cell type	11	12	23	13
7	Spindle cell type	10	11	14	12
8	Mixed cell type	10	10	14	12
9	Spindle cell type	4	3	14	16
10	Mixed cell type	11	10	12	10.5
11	Spindle cell type	7	10	18	15
12	Mixed cell type	11	10	16	12
13	Mixed cell type	14	7	14	12
14	Mixed cell type	8	10	15	20
15	Spindle cell type	14	7	15	17
16	Spindle cell type	9	7	12	9
17	Epithelioid cell type	10	10	19	15
18	Mixed cell type	7	5	16.5	15
19	Epithelioid cell type	12	11	10	12
20	Spindle cell type	14	13	19	16
21	Mixed cell type	12	11	19	17
22	Spindle cell type	10	10	12.5	11
23	Mixed cell type	5	4	12	15
24	Mixed cell type	15	10	18	18
25	Spindle cell type	9	6	12.5	10
26	Mixed cell type	10	12	15	15

* Measured including the scleral thickness.

## Data Availability

The data presented in this study are available on request from the corresponding author.
